# The Pseudomonas aeruginosa Complement of Lactate Dehydrogenases Enables Use of d- and l-Lactate and Metabolic Cross-Feeding

**DOI:** 10.1128/mBio.00961-18

**Published:** 2018-09-11

**Authors:** Yu-Cheng Lin, William Cole Cornell, Jeanyoung Jo, Alexa Price-Whelan, Lars E. P. Dietrich

**Affiliations:** a Department of Biological Sciences, Columbia University, New York, New York, USA; Emory University School of Medicine

**Keywords:** biofilms, lactate isomers, pyruvate, pyruvate fermentation

## Abstract

Pseudomonas aeruginosa is the most common cause of chronic, biofilm-based lung infections in patients with cystic fibrosis (CF). Sputum from patients with CF has been shown to contain oxic and hypoxic subzones as well as millimolar concentrations of lactate. Here, we describe the physiological roles and expression patterns of P. aeruginosa lactate dehydrogenases in the contexts of different growth regimes. P. aeruginosa produces four enzymes annotated as lactate dehydrogenases, three of which are known to contribute to anaerobic or aerobic metabolism in liquid cultures. These three are LdhA, which reduces pyruvate to d-lactate during anaerobic survival, and LldE and LldD, which oxidize d-lactate and l-lactate, respectively, during aerobic growth. We demonstrate that the fourth enzyme, LldA, performs redundant l-lactate oxidation during growth in aerobic cultures in both a defined MOPS (morpholinepropanesulfonic acid)-based medium and synthetic CF sputum media. However, LldA differs from LldD in that its expression is induced specifically by the l-enantiomer of lactate. We also show that the P. aeruginosa lactate dehydrogenases perform functions in colony biofilms that are similar to their functions in liquid cultures. Finally, we provide evidence that the enzymes LdhA and LldE have the potential to support metabolic cross-feeding in biofilms, where LdhA can catalyze the production of d-lactate in the anaerobic zone, which is then used as a substrate in the aerobic zone. Together, these observations further our understanding of the metabolic pathways that can contribute to P. aeruginosa growth and survival during CF lung infection.

## Abstract (precis)

Lactate is thought to serve as a carbon and energy source during chronic infections. Sites of bacterial colonization can contain two enantiomers of lactate: the l-form, generally produced by the host, and the d-form, which is usually produced by bacteria, including the pulmonary pathogen Pseudomonas aeruginosa. Here, we characterize P. aeruginosa’s set of four enzymes that it can use to interconvert pyruvate and lactate, the functions of which depend on the availability of oxygen and specific enantiomers of lactate. We also show that anaerobic pyruvate fermentation triggers production of the aerobic d-lactate dehydrogenase in both liquid cultures and biofilms, thereby enabling metabolic cross-feeding of lactate over time and space between subpopulations of cells. These metabolic pathways might contribute to P. aeruginosa growth and survival in the lung.

## INTRODUCTION

During growth and survival in communities, bacteria encounter microniches with conditions that differ from those of the external environment. Gradients form over biofilm depth due to consumption of resources by cells closer to the periphery (1–3). Efforts to control biofilm behavior in clinical and industrial settings depend on our understanding of the physiological responses to these unique conditions.

We study the effects of biofilm resource gradients on the physiology of the opportunistic pathogen Pseudomonas aeruginosa, a major cause of biofilm-based infections and the most prominent cause of lung infections in patients with the inherited disease cystic fibrosis (CF) (4). P. aeruginosa can generate ATP for growth via oxygen and nitrate respiration and, to a limited extent, through arginine fermentation (5, 6). Sufficient ATP to support survival can be generated via (i) pyruvate fermentation (7) or (ii) cyclic reduction of endogenously produced antibiotics called phenazines, under conditions in which these compounds are reoxidized outside the cell (8, 9). We have found that some of these pathways for ATP generation also facilitate redox balancing for cells in biofilms and that P. aeruginosa modulates its overall community architecture in response to the availability of oxygen, nitrate, and phenazines (1, 10).

Previous work has indicated that phenazine-supported ATP generation and survival in anaerobic cell suspensions depend on reactions associated with pyruvate fermentation and oxidation (7, 9). Furthermore, lactate, a product of pyruvate fermentation, is a major component of CF sputum (11) and a significant carbon and energy source for pathogens and commensals of mammalian hosts (12–15). These observations motivated us to investigate the roles of enzymes that interconvert pyruvate and lactate in P. aeruginosa growth and biofilm development, including a previously uncharacterized l-lactate dehydrogenase. We found that this enzyme plays a redundant role in aerobic growth on l-lactate but that its expression is uniquely and specifically induced by the l-enantiomer of lactate, which is typically produced by plant and mammalian metabolism (16–18). Our studies also show that biofilms grown on pyruvate have the potential to engage in substrate cross-feeding, in which d-lactate produced fermentatively in the anoxic microniche acts as the electron donor for aerobic respiration in the upper, oxic portion of the biofilm. These results help us to further define potential pathways of electron flow in P. aeruginosa biofilm cells and the diverse metabolisms that might operate simultaneously in bacterial communities.

## RESULTS AND DISCUSSION

### The P. aeruginosa genome encodes four enzymes that interconvert pyruvate and lactate.

**FIG 1:**
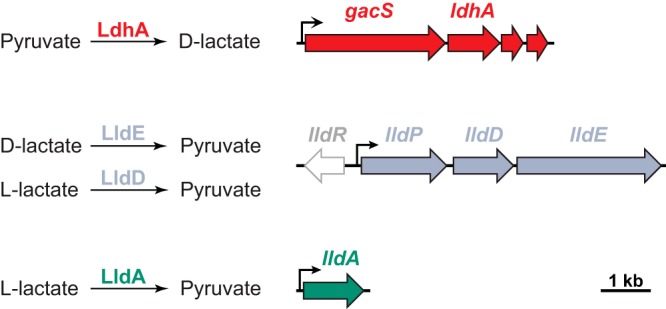
The P. aeruginosa genome encodes several enzymes that interconvert pyruvate and lactate. (Left) Reactions catalyzed by P. aeruginosa’s lactate dehydrogenases; (right) chromosomal loci encoding each of the corresponding enzymes. LdhA catalyzes the reduction of pyruvate during anaerobic survival. LldE catalyzes the oxidation of d-lactate during aerobic growth. Unlike E. coli, which contains only one gene encoding an l-lactate dehydrogenase, P. aeruginosa contains two orthologues for this enzyme. LldD catalyzes the oxidation of l-lactate during aerobic growth. This study describes a role for LldA in catalyzing the oxidation of l-lactate during aerobic growth.

To initiate our characterization of pathways for pyruvate and lactate utilization in P. aeruginosa PA14, we examined the genome for loci encoding lactate dehydrogenases. PA14 contains four genes with the following annotation: *ldhA* (PA14_52270), *lldD* (PA14_63090), *lldE* (PA14_63100), and *lldA* (PA14_33860) (Fig. 1). *ldhA* encodes a lactate dehydrogenase that reduces pyruvate to lactate during anaerobic pyruvate fermentation (7) (Fig. 1). According to computational prediction (19), *ldhA* is cotranscribed with three other genes, including that encoding the global regulator GacS. *lldD* and *lldE* encode an l-lactate dehydrogenase and a d-lactate dehydrogenase, respectively, and are cotranscribed with *lldP*, which encodes a lactate permease. *lldR*, which encodes a repressor of *lldPDE* expression that is deactivated by either l- or d-lactate, lies adjacent to the *lldPDE* operon and is divergently transcribed (Fig. 1) (20). P. aeruginosa PA14 encodes a second, uncharacterized l-lactate dehydrogenase, called LldA, that is 44% identical to LldD. Though few other pseudomonad species contain more than one l-lactate dehydrogenase (see Fig. S1A to C in the supplemental material) (19), the P. aeruginosa arrangement of *lldR* and *lldPDE* (Fig. S1B) is common within the *Pseudomonas* genus. In the model organism Escherichia coli, by contrast, the *lldPRD* genes are arranged in an operon and *lldR* responds specifically to l-lactate (21), while the E. coli d-lactate dehydrogenase Dld is encoded separately (Fig. S1D) (22).

10.1128/mBio.00961-18.1 FIG S1 Operon structures and distribution of lactate dehydrogenases in selected species of interest. (A) Schematic of reactions catalyzed by lactate dehydrogenases and color key for panels B to E. (B) Genomic arrangement and operon structures for loci containing *lldD*, *lldE*, and *lldA* for representative pseudomonad species. (C) Distribution of *lldE*, *lldD*, and *lldA* in *Pseudomonas* species (data were retrieved from the Pseudomonas Genome Database). (D) Genomic arrangement and operon structures for loci containing orthologs of l-lactate dehydrogenase genes in E. coli and four common CF pathogens. (E) Phylogenetic tree of lactate dehydrogenase sequences found in P. aeruginosa and the species shown in panel D. Proteins were aligned using ClustalW (BLOSUM), and the tree was generated using the Jukes-Cantor genetic distance model and the neighbor-joining tree build method. Download FIG S1, EPS file, 1.6 MB. Copyright © 2018 Lin et al. 2018 Lin et al. This content is distributed under the terms of the Creative Commons Attribution 4.0 International license.

### The *lldPDE* and *lldA* loci are induced by substrates of their respective protein products.

Upon noticing the second gene encoding an l-lactate dehydrogenase (i.e., LldA) in the P. aeruginosa genome, we wondered whether we could observe its expression under conditions similar to those that induce the expression of LldD. We therefore created a suite of P. aeruginosa PA14 strains that express green fluorescent protein (GFP) under the control of putative promoters for lactate dehydrogenase genes. For the *lldPDE* and *lldA* reporters, we treated ∼300 bp of sequence upstream of each of these loci as putative promoters. However, we viewed *ldhA* as a unique case because it was computationally predicted to be cotranscribed with *gacS*. GacS is a notorious upstream regulator of quorum sensing (23) and therefore plays a role in P. aeruginosa physiology distinct from that of *ldhA.* We examined transcriptome sequencing (RNA-seq) data obtained from PA14 biofilms to identify transcriptional start sites in the *gacS*-*ldhA* region of the genome. This profiling showed a transcriptional start site at ∼46 bp upstream of the start codon of *gacS* and steady numbers of reads extending through *ldhA* (Fig. S2A), suggesting that *ldhA* expression is driven by the promoter upstream of *gacS*. Nevertheless, we created reporter strains that contained portions of sequence from the regions upstream of either *gacS* or *ldhA*, respectively, so that we could detect any potential, independent expression of *ldhA*.

10.1128/mBio.00961-18.2 FIG S2 *gacS* and *ldhA* are cotranscribed. (A) RNA-seq read profile at the *gacS*-*ldhA* locus. The *y* axis represents read coverage for each nucleotide position in the genome. The coding sequences are represented by horizontal arrows. A transcriptional start site (TSS) was observed at ∼46 bp upstream of the *gacS* coding sequence. (B) Strains engineered to express GFP under the control of promoters upstream of *gacS* or *ldhA* were grown in MOPS medium, with the indicated compounds provided as sole carbon sources. Error bars, which are obscured by the point marker in most cases, represent the standard deviations from biological triplicates. Download FIG S2, EPS file, 1.8 MB. Copyright © 2018 Lin et al. 2018 Lin et al. This content is distributed under the terms of the Creative Commons Attribution 4.0 International license.

**FIG 2:**
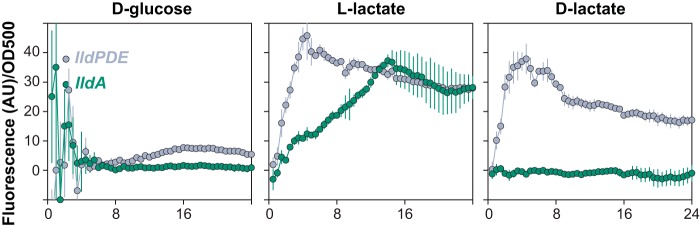
Expression of loci associated with pyruvate and lactate metabolism during aerobic, liquid-culture growth. Strains engineered to express GFP under the control of promoters upstream of *lldP* (which is cotranscribed with *lldD* and *lldE*) or *lldA* were grown in MOPS medium, with the indicated compounds provided as sole carbon sources. Background fluorescence from a strain with a promoterless reporter was subtracted before normalization to the OD at 500 nm. Error bars, which are often obscured by the point markers, represent the standard deviations from biological triplicates. AU, arbitrary units.

The reporter strains were grown aerobically in defined media. We observed very low levels of expression from the *gacS* promoter, but not the putative *ldhA* promoter region, as predicted from the RNA-seq profiling (Fig. S2B). Cotranscription of *gacS* and *ldhA* makes physiological sense in that the high cell density associated with quorum sensing leads to oxygen limitation and therefore a condition in which cells potentially benefit from pyruvate fermentation. A low level of expression was also observed after ∼9 h of growth for *lldPDE*, but not *lldA*, on d-glucose (left panel of Fig. 2). *lldPDE* expression was strongly induced from the start of growth and peaked around 4 h, when either d- or l-lactate was provided as the sole carbon source (middle and right panels of Fig. 2). These results are consistent with a previous study of P. aeruginosa XMG (20), which showed that LldR responds to both enantiomers of lactate. In contrast, *lldA* expression was not observed on d-glucose or d-lactate and was induced strictly by l-lactate (Fig. 2). This finding is consistent with LldA’s predicted physiological function and indicates that it is controlled by a regulator that specifically senses the l-enantiomer. In contrast to that of *lldPDE*, *lldA* expression showed a more gradual increase over the first several hours of growth and peaked at around 14 h.

### Both LldD and LldA contribute to l-lactate utilization during liquid-culture and biofilm growth.

**FIG 3:**
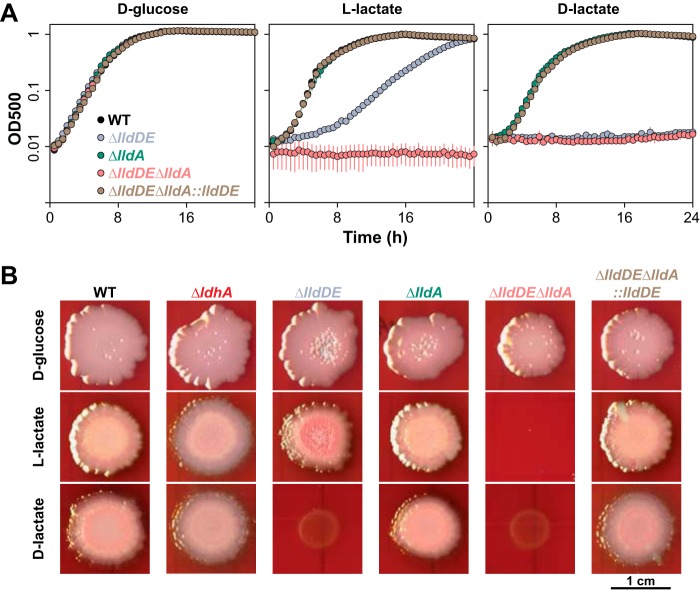
Physiological roles of enzymes that interconvert pyruvate and lactate during growth in shaken liquid cultures and biofilms. (A) Aerobic growth of the indicated strains in MOPS medium with d-glucose, l-lactate, or d-lactate provided as the sole carbon source. Error bars, which are obscured by the point marker in most cases, represent the standard deviations from biological triplicates. (B) Growth and morphological development of the indicated strains under an oxic atmosphere on MOPS medium containing the dyes Congo red and Coomassie blue and amended with d-glucose, l-lactate, or d-lactate. Images were taken after 4 days of incubation. WT, wild type.

To test whether LldA contributes uniquely to l-lactate utilization, we generated deletion strains lacking *lldDE*, *lldA*, or both loci and tested their abilities to grow aerobically in defined media. When l-lactate was provided as the sole carbon source, deletion of *lldDE* did not completely abolish growth but rather led to biphasic growth at lower rates than that observed for the wild type (Fig. 3A). This slow growth of the Δ*lldDE* mutant on l-lactate contrasts with that seen for an equivalent mutant created in Pseudomonas stutzeri SDM, which shows no growth on l-lactate (24), and indicates that LldD is not the only enzyme that oxidizes l-lactate in P. aeruginosa. Indeed, deletion of *lldA* in the Δ*lldDE* background yielded a strain that was completely defective in growth on l-lactate (Fig. 3A). However, the Δ*lldA* single deletion did not result in any growth defect on l-lactate (Fig. 3A), suggesting that LldA’s activity is redundant with that of LldD. Consistent with findings reported for P. stutzeri SDM (24), we found that *lldDE* was necessary for growth with d-lactate (Fig. 3A), indicating that LldE is the only enzyme that oxidizes this enantiomer of lactate during aerobic growth.

The d- and l-enantiomers of lactate are generally associated with bacterial and metazoan metabolism, respectively, with l-lactate being the primary form produced in sites of microbial colonization, such as the mammalian gut (13) and the airways of patients with CF (25). We were therefore interested in the relevance of lactate dehydrogenase activity to the growth and morphogenesis of biofilms, which represent a major lifestyle assumed by commensal and pathogenic bacteria. To examine this, we grew deletion mutants lacking lactate dehydrogenase genes as colony biofilms on defined media containing the dyes Congo red and Coomassie blue, which aid visualization of morphogenetic features. Generally, the colony biofilm phenotypes of the mutants matched phenotypes observed during aerobic growth in liquid cultures (Fig. 3B). The lack of an effect of *ldhA* deletion (Fig. 3B) under these conditions is consistent with the role of LdhA as a pyruvate reductase (7). We note that, in addition to having its expected, moderate growth defect on l-lactate, the Δ*lldDE* mutant showed increased production of matrix (as indicated by increased binding of Congo red) (Fig. 3B). In other work (1), we have found that this phenotype often correlates with impaired redox balancing and a reduced cellular redox state; in this case, therefore, it might indicate that the LldA enzyme does not sufficiently support the maintenance of redox homeostasis in colony biofilms.

### The l-lactate in synthetic cystic fibrosis media contributes to PA14 growth.

**FIG 4:**
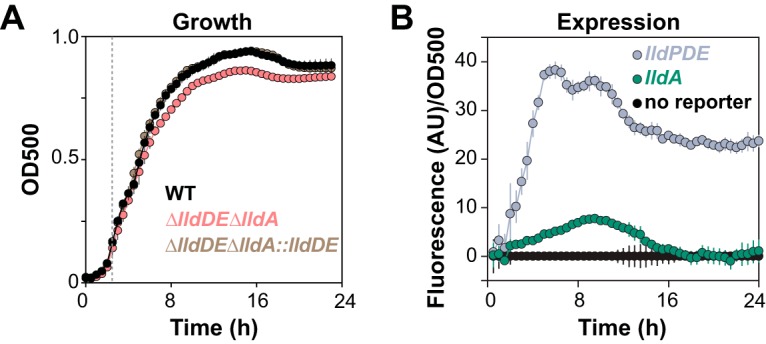
PA14 utilizes the l-lactate in synthetic cystic fibrosis sputum medium (SCFM) for growth. (A) Growth of the indicated strains in SCFM. Error bars represent the standard deviations from at least four biological replicates and are omitted in cases where they would be obscured by point markers. *P* values of the Δ*lldDE* Δ*lldA* double mutant versus the wild type and Δ*lldDE* Δ*lldA*::*lldDE* strain are <0.0001 based on an unpaired, two-tailed *t* test. (B) Expression of the indicated reporter constructs in SCFM. Background fluorescence from a strain with a promoterless reporter was subtracted before normalization to the OD at 500 nm.

Lactate is a major component of CF sputum (11), and its concentration correlates with the exacerbation of chronic infections in CF lungs (25). We predicted that LldD and/or LldA would contribute to PA14 growth in synthetic CF sputum media (11, 26, 27), which contain millimolar concentrations of l-lactate. We grew mutants lacking these l-lactate dehydrogenases in three different media that imitate nutrient availability in CF lungs: (i) synthetic CF sputum medium (SCFM), (ii) modified artificial sputum medium (ASMDM, which is similar to SCFM but also contains bovine serum albumin [BSA], mucin, and herring sperm DNA), and (iii) SCFM2, which is similar to ASMDM but contains additional large molecules. We found that the Δ*lldDE* Δ*lldA* double mutant, which is not able to grow using l-lactate as a sole carbon source (Fig. 3), showed an ∼10% decrease in growth (Fig. 4A) in SCFM, which was evident after cultures entered late stationary phase (Fig. 4A and S3). Both *lldPDE* and *lldA* were transcriptionally induced in SCFM, though *lldPDE* was expressed at a higher level than *lldA* (Fig. 4B). We also detected a marked defect for the double mutant in ASMDM, though interestingly, it occurred earlier in the growth curve (Fig. S3). A modest defect was also detectable in SCFM2 (Fig. S3). These results suggest that LldD and LldA together might contribute to l-lactate utilization for growth in the CF lung environment. Our observations indicate that lactate dehydrogenases make less significant contributions to growth in a more complex medium containing some of the large molecules and polymers available in this setting; however, the CF lung environment is heterogeneous and might nevertheless contain subregions where lactate is a major source of carbon and energy.

10.1128/mBio.00961-18.3 FIG S3 Growth in SCFM, ASMDM, and SCFM2. Growth of the indicated strains in synthetic media designed to mimic nutrient availability in the CF lung environment. Each data set is plotted on linear (left panels) and logarithmic (right panels) scales. Data represent the means from at least four biological replicates. Error bars represent standard deviations and are not shown where obscured by point markers. Download FIG S3, EPS file, 2.1 MB. Copyright © 2018 Lin et al. 2018 Lin et al. This content is distributed under the terms of the Creative Commons Attribution 4.0 International license.

### LldE supports the growth of PA14 on self-produced d-lactate.

**FIG 5:**
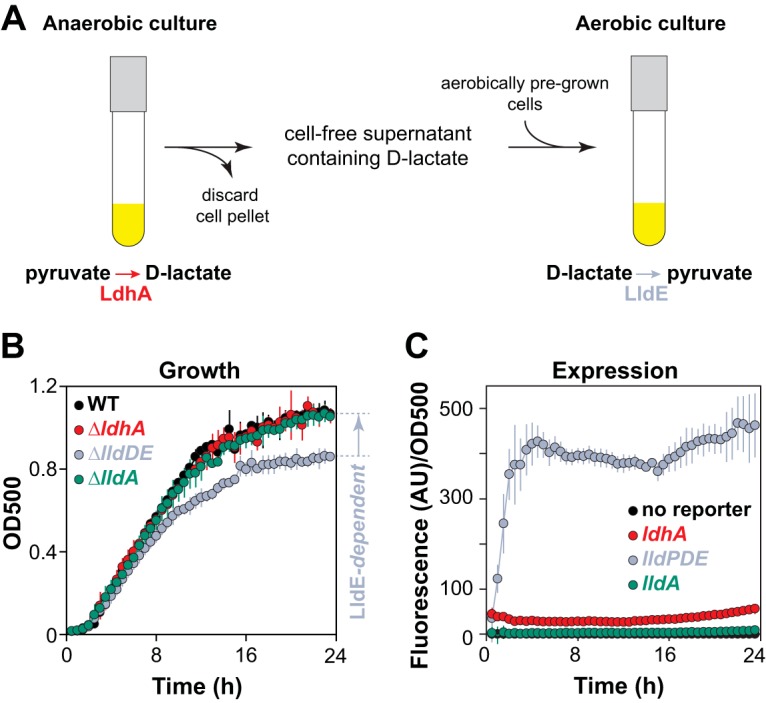
Growth of PA14 on self-produced d-lactate. (A) Design schematic for a pyruvate/d-lactate cross-feeding experiment in liquid cultures. P. aeruginosa PA14 was incubated in anoxic liquid medium (MOPS plus 0.1% tryptone plus 40 mM sodium pyruvate) to promote the fermentation and production of d-lactate. The supernatants collected from these cultures were used as the growth medium for oxic cultures, in which the d-lactate then served as a carbon source for PA14 growth. (B) Growth of the indicated strains on supernatants obtained from anaerobic cultures that had fermented pyruvate. Error bars represent the standard deviations from three biological replicates. *P* values for the Δ*lldDE* mutant versus the wild type, Δ*lldA* mutant, and Δ*ldhA* mutant are 4.6 × 10^−4^, 2.2 × 10^−4^, and 1.1 × 10^−3^, respectively, and are based on unpaired, two-tailed *t* test results with equal variances. (C) Expression of the indicated reporter constructs during growth on the supernatant described in panel A. Background fluorescence from a strain with a promoterless reporter was subtracted before normalization to the OD at 500 nm.

The fact that the P. aeruginosa genome encodes enzymes that catalyze the oxygen-dependent interconversion of pyruvate and d-lactate raises the possibility that cells in environments where oxygen availability varies over time or space engage in metabolic cross-feeding. In this scenario, pyruvate is reduced to d-lactate under anoxic conditions, and then d-lactate is oxidized to pyruvate and further metabolized under oxic conditions. We set out to test this possibility in liquid cultures by first incubating P. aeruginosa anaerobically with pyruvate (to stimulate d-lactate production) and then using the supernatant from these cultures as the medium for aerobic growth of fresh cultures (which should use the d-lactate in an *lldE*-dependent manner) (Fig. 5A). First, we verified that *ldhA* supports the survival of PA14 under anaerobic conditions in which pyruvate is provided as the major carbon source (Fig. S4). We found that this phenomenon required the inclusion of a complex nutrient source (e.g., lysogeny broth [LB] or tryptone) in the medium, possibly as a source of an unidentified cofactor used in pyruvate fermentation. We therefore carried out a series of titration experiments in order to identify a concentration of tryptone that would allow us to observe *lldE*-dependent growth (Fig. S5A) and *lldPDE* expression (Fig. S5B) at concentrations of lactate that could be produced in the pyruvate fermentation cultures (7). Next, we incubated PA14 cells for 7 days in anoxic liquid 0.1% tryptone medium containing 40 mM pyruvate, collected cell-free spent medium from these cultures, and used it as the growth medium for freshly inoculated aerobic cultures (Fig. 5A). Consistent with the model in which PA14-generated d-lactate was present in the medium and acted as a carbon source (Fig. 5A), this experimental design yielded high levels of expression from the *lldPDE* reporter (Fig. 5C) and revealed a growth defect for the Δ*lldDE* mutant (Fig. 5B).

10.1128/mBio.00961-18.4 FIG S4 Long-term anaerobic survival. Anaerobic survival of the indicated strains in phosphate-buffered LB medium with or without pyruvate. Error bars represent the standard deviations of results from biological triplicates. Download FIG S4, EPS file, 1.2 MB. Copyright © 2018 Lin et al. 2018 Lin et al. This content is distributed under the terms of the Creative Commons Attribution 4.0 International license.

10.1128/mBio.00961-18.5 FIG S5 Liquid-culture growth with varied concentrations of tryptone and d-lactate. (A) Growth of the wild type and Δ*lldDE* mutant. For results in the top panel, d-lactate was set at 4 mM and tryptone was varied from 0% to 0.5%. For results in the bottom panel, tryptone was set at 0.5% and d-lactate was varied from 0 to 40 mM. (B) Expression of the *lldPDE* reporter construct in the same media used in panel A. Media were prepared with MOPS buffer. Error bars represent the standard deviations of results from biological triplicates. Download FIG S5, EPS file, 2.2 MB. Copyright © 2018 Lin et al. 2018 Lin et al. This content is distributed under the terms of the Creative Commons Attribution 4.0 International license.

**FIG 6:**
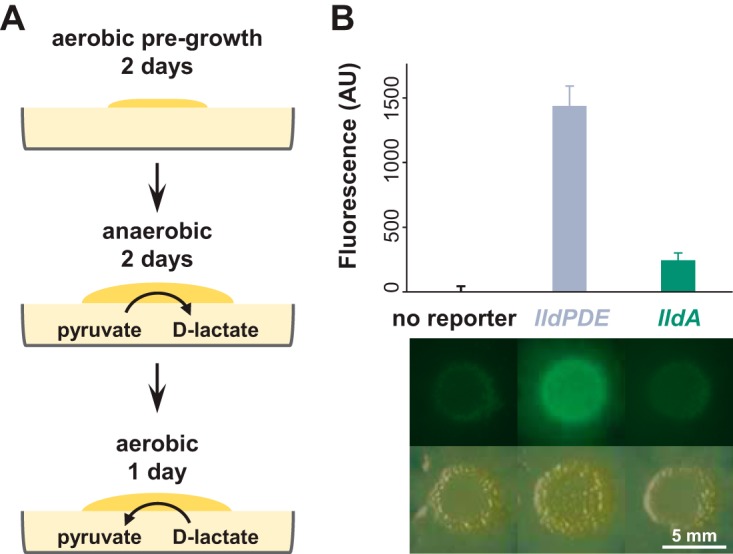
Self-produced d-lactate induces *lldPDE* in biofilms. (A) Design schematic for a pyruvate/d-lactate cross-feeding experiment in liquid cultures in colony biofilms. Colonies were initially grown atop filter membranes on plates containing 1% tryptone, 1% agar plus 40 mM sodium pyruvate for 2 days under an oxic atmosphere to establish biomass and then transferred to fresh plates of medium containing 0.1% tryptone, 1% agar plus 40 mM sodium pyruvate in an anoxic chamber to stimulate pyruvate fermentation and d-lactate production for 2 days. Finally, the plates were moved from the chamber and back into an oxic atmosphere for 1 day. The procedure was carried out at room temperature. (B) Fluorescence quantification (top) and images (bottom) of colonies grown using the procedure shown in panel A. Background fluorescence was normalized to the “no reporter” control. Error bars represent the standard deviations from three biological replicates. *P* values for lldPDE reporter versus no reporter and lldA reporter are 1.0 × 10^−4^ and 2.3 × 10^−4^, respectively, and are based on unpaired, two-tailed *t* test results with equal variance.

We next asked whether PA14 has the potential to utilize self-produced d-lactate during growth in colony biofilms. Colony biofilms were first grown aerobically on pyruvate for 2 days to establish sufficient biomass and then transferred to fresh anoxic plates for 2 days to promote pyruvate fermentation and d-lactate production. These plates were then subsequently moved to oxic conditions and incubated for 1 day (Fig. 6A). This procedure yielded high expression of the *lldPDE* reporter (controlled by an l- and d-lactate sensor) but not the *lldA* reporter (controlled by an l-lactate-specific sensor) (Fig. 6B), indicating the presence of self-produced d-lactate, which might potentially be utilized by PA14 in colony biofilms.

### P. aeruginosa PA14 cells in biofilms have the potential to engage in pyruvate/lactate cross-feeding over the oxygen gradient.

**FIG 7:**
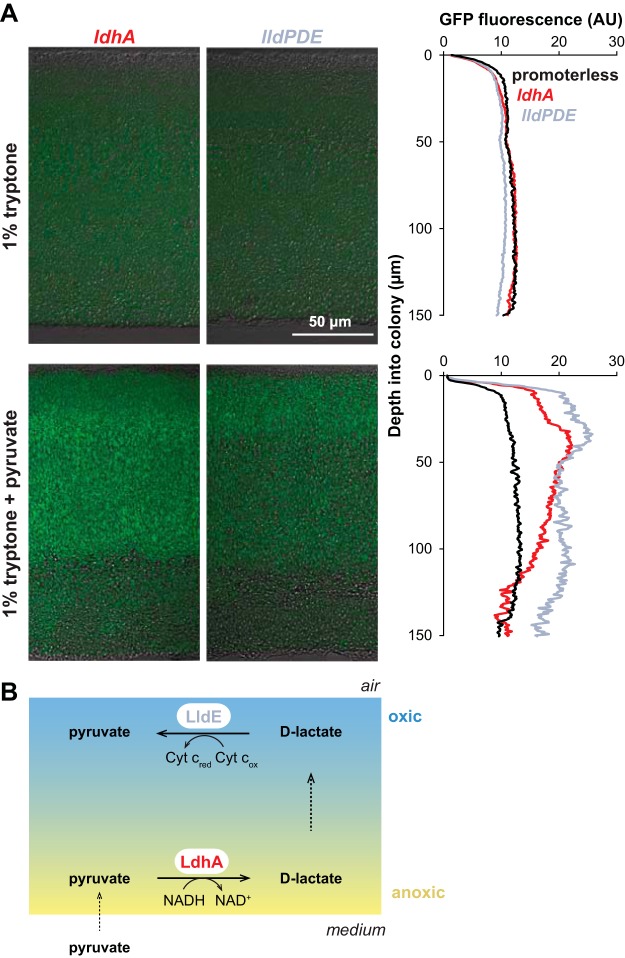
Expression of enzymes for interconversion of pyruvate and lactate over biofilm depth. Expression of *ldhA* (which catalyzes pyruvate reduction under anaerobic conditions) and *lldE* (which catalyzes d-lactate oxidation under aerobic conditions) as reported by promoter-*gfp* fusions in thin sections from wild-type PA14 biofilms. Biofilms were grown for 3 days on 1% tryptone medium with or without 10 mM pyruvate. Quantification of fluorescence over depth is shown at the right. This experiment was repeated in biological triplicate, and representative results are shown. Cyt c_red_, with reduced cytochrome *c*; Cyt c_ox_, with oxidized cytochrome *c*.

As PA14 colony biofilms increase in thickness, they develop steep oxygen gradients, leading to the formation of metabolic subpopulations in oxic and anoxic zones (28). After observing that PA14 could produce d-lactate under fermentative conditions that acts as an electron donor for aerobic growth, we hypothesized that pyruvate fermentation and aerobic d-lactate utilization cooccur in biofilms. To test this, we grew colony biofilms on medium with and without pyruvate and examined the expression of the *lldPDE* operon, which is induced by d-lactate. We observed higher expression of *lldPDE* when colonies were grown on medium containing pyruvate (Fig. 7A), indicating that cells in the anoxic zone carried out pyruvate fermentation and produced d-lactate. These results suggest that, in addition to generating ATP and supporting survival for cells in oxygen-limited regions of biofilms, pyruvate fermentation can serve to produce d-lactate that supports growth in regions where oxygen is available (Fig. 7B). Overall, they add a layer of complexity to our picture of the integrated metabolisms occurring in physiologically heterogeneous bacterial communities.

### Concluding remarks.

Metabolic cross-feeding has been described for polymicrobial communities that are models for those found within the human body and that colonize sites such as the oral cavity and large intestine (29, 30). Our findings indicate that metabolic cross-feeding might take place in a single-species biofilm and arise from physiological heterogeneity, in which a fermentation product formed in the anaerobic microniche is oxidized by cells in the aerobic microniche. Our model for this cross-feeding in biofilms might be viewed as a form of electron shuttling (Fig. 7B), with the role of d-lactate as an electron carrier between anoxic and oxic subzones showing some similarities to that of phenazines (1, 8, 28).

Most pseudomonad species have either *lldD* or *lldA* for l-lactate utilization (Fig. S1C). We have shown that in P. aeruginosa PA14, *lldD* and the previously uncharacterized orphan *lldA* encode l-lactate dehydrogenases with redundant functions during growth in liquid cultures. However, *lldA* differs from *lldD* in that it is specifically induced by l-lactate. The presence of redundant l-lactate dehydrogenases in P. aeruginosa may represent an adaptation to the host environment, given the fact that host organisms, such as humans and plants (18, 31, 32), produce primarily the l-enantiomer. Several studies have implicated l-lactate metabolism in bacterial virulence during infections, including *Salmonella*-induced gastroenteritis (13) and gonococcal infection of the genital tract (12). Our results suggest that LldD and LldA utilize l-lactate in SCFM and other artificial sputum media to promote PA14 growth (Fig. 4A and Fig. S3). Similarly, other common CF pathogens encode l-lactate dehydrogenases homologous to LldD and LldA. Using the P. aeruginosa PA14 LldD, LldA, and LldE protein sequences, we searched for homologues in representative strains of Achromobacter xylosoxidans, Burkholderia multivorans, Haemophilus influenzae, and Stenotrophomonas maltophilia (33–35). We found that H. influenzae and S. maltophilia contain an l-lactate dehydrogenase homolog but that A. xylosoxidans and B. multivorans, like P. aeruginosa, contain multiple homologs. As l-lactate is a significant metabolite available in the lungs of CF patients (11, 25, 36, 37), these enzymes may contribute to the abilities of P. aeruginosa and other pathogens to colonize and persist in this environment.

## MATERIALS AND METHODS

### Bacterial strains and growth conditions.

Unless otherwise indicated, P. aeruginosa strain UCBPP-PA14 and mutants thereof (38) were routinely grown in lysogeny broth (LB; 1% tryptone, 1% NaCl, 0.5% yeast extract) (39) at 37°C with shaking at 250 rpm. Overnight cultures were grown for 16 ± 1 h. For genetic manipulation, strains were typically grown on LB solidified with 1.5% agar. Strains used in this study are listed in Table S1 in the supplemental material. In general, liquid precultures served as inocula for experiments. Overnight precultures for biological replicates were started from separate clonal colonies on streak plates.

10.1128/mBio.00961-18.6 TABLE S1 Strains and plasmids used in this study. Download Table S1, PDF file, 0.3 MB. Copyright © 2018 Lin et al. 2018 Lin et al. This content is distributed under the terms of the Creative Commons Attribution 4.0 International license.

### Construction of mutant P. aeruginosa strains.

For making markerless deletion mutants in P. aeruginosa PA14 (Table S1), ∼1-kb flanking sequences from each side of the target gene were amplified using the primers listed in Table S2 and inserted into the allelic-replacement vector pMQ30 through gap repair cloning in Saccharomyces cerevisiae InvSc1 (40). Each plasmid listed in Table S1 was transformed into Escherichia coli strain UQ950, verified by sequencing, and moved into PA14 using biparental conjugation. PA14 single recombinants were selected on LB agar plates containing 100 μg/ml gentamicin. Double recombinants (markerless deletions) were selected on sucrose plates (1% tryptone, 0.5% yeast extract, 10% sucrose, and 1.5% agar). Genotypes of deletion mutants were verified by PCR. Combinatorial mutants were constructed by using single mutants as parent strains.

10.1128/mBio.00961-18.7 TABLE S2 Primers used in this study. Download Table S2, PDF file, 0.1 MB. Copyright © 2018 Lin et al. 2018 Lin et al. This content is distributed under the terms of the Creative Commons Attribution 4.0 International license.

### Construction of GFP reporter strains.

Transcriptional reporter constructs for the genes *ldhA*, *gacS*, *lldP*, and *lldA* were made by fusing their promoter sequences with *gfp* using primers listed in Table S2. Respective primers were used to amplify promoter regions (as indicated in Table S1) and to add an SpeI digest site to the 5′ end of the promoter and an XhoI digest site to its 3′ end. For the *ldhA* reporter, an EcoRI site was used instead of XhoI. Purified PCR products were digested and ligated into the multiple-cloning site (MCS) upstream of the *gfp* sequence of pLD2722, which is a derivative of pYL122 (41) and contains a ribosome-binding site between the MCS and *gfp*. Plasmids were transformed into E. coli strain UQ950, verified by sequencing, and moved into PA14 using biparental conjugation. Conjugative transfer of pLD2722 was conducted with the E. coli *strain* S17-1 (41). PA14 single recombinants were selected on M9 minimal medium agar plates (47.8 mM Na_2_HPO_4_, 22 mM KH_2_PO_4_, 8.6 mM NaCl, 18.6 mM NH_4_Cl, 1 mM MgSO_4_, 0.1 mM CaCl_2_, 20 mM sodium citrate dihydrate, 1.5% agar) containing 70 μg/ml gentamicin. The plasmid backbone of pLD2722 was resolved from PA14 using Flp-Flp recombination target (FRT) recombination by introduction of the pFLP2 plasmid (42) and selected on M9 minimal medium agar plates containing 300 μg/ml carbenicillin and further on sucrose plates (1% tryptone, 0.5% yeast extract, 10% sucrose, 1.5% agar). The presence of *gfp* in the final clones was confirmed by PCR.

### Liquid-culture growth assays.

Overnight (16-h) precultures were diluted 1:100 in a clear-bottom, polystyrene black 96-well plate (VWR 82050-756), with each well containing 200 μl of medium. Cultures were then incubated at 37°C with continuous shaking at medium speed in a BioTek Synergy 4 plate reader. Reporter strains were grown in MOPS medium (50 mM MOPS, 43 mM NaCl, 93 mM NH_4_Cl, 2.2 mM KH_2_PO_4_, 1 mM MgSO_4_, 1 μg/ml FeSO_4_ at pH 7.0) amended with one of the following carbon sources: 20 mM d-glucose, 40 mM l-lactate, or 40 mM d-lactate (Sigma-Aldrich). Expression of GFP was assessed by taking fluorescence readings at excitation and emission wavelengths of 480 nm and 510 nm, respectively, every 30 min for up to 24 h. Growth was assessed by taking readings of optical density at 500 nm (OD_500_) simultaneously with the fluorescence readings.

### Colony growth assays.

Overnight (16-h) precultures were diluted 1:10 in phosphate-buffered saline (PBS). Five microliters of diluted cultures was spotted onto MOPS medium (50 mM MOPS, 43 mM NaCl, 93 mM NH_4_Cl, 2.2 mM KH_2_PO_4_, 1 mM MgSO_4_, 1 μg/ml FeSO_4_ at pH 7.0) amended with one of the following carbon sources: 20 mM d-glucose, 40 mM l-lactate, or 40 mM d-lactate (Sigma-Aldrich). The culture medium was then solidified with 1% agar (Teknova). Colonies were incubated at 25°C for up to 4 days and imaged with an Epson Expression 11000XL scanner. Fluorescence images (Fig. 6B) were taken with a Zeiss Axio Zoom.V16 microscope (excitation, 488 nm; emission, 509 nm for imaging of GFP fluorescence).

### Anaerobic survival assays.

For each anaerobic liquid sample, 50 μl of overnight (16-h) precultures were diluted in 5 ml of potassium phosphate (100 mM)-buffered (pH 7.4) LB (7, 43) in a Balch tube and then incubated at 37°C with shaking at 250 rpm for 2.5 h to early to mid-exponential phase (OD_500_, ∼0.5). Balch tubes containing subcultures were then transferred into the anaerobic chamber (80% N_2_, 15% CO_2_, and 5% H_2_) and sealed with rubber stoppers to maintain anoxia. Anaerobic tubes were incubated with shaking at 250 rpm at 37°C throughout the period of the survival assay. To measure CFU from the anaerobic culture, 10 to 50 μl of culture was aspirated from the tube with a 1-ml syringe (Care Touch) fitted with a 23-gauge needle (Becton, Dickinson), and then serially diluted in LB down to 10^−6^ CFU and plated on 1% tryptone plates for CFU counting.

### Thin sectioning and preparation for microscopic analyses.

Thin sections of P. aeruginosa colonies were prepared as described previously (44). Briefly, to produce bilayer plates, a 4.5-mm-thick bottom layer of medium (1% agar,1% tryptone) was poured and allowed to solidify before a 1.5-mm-thick top layer was poured. Precultures were incubated overnight, diluted 1:100 in LB, and incubated until early to mid-exponential phase (OD_500_, ∼0.5). Five microliters of culture were spotted onto the top agar layer and incubated in the dark at 25°C with >90% humidity (Percival CU-22L) for up to 3 days. Colonies were sacrificed for thin sectioning at specified time points by first covering them with a 1.5-mm-thick layer of 1% agar, which sandwiches each colony between two 1.5-mm layers of solidified agar. Sandwiched colonies were lifted from the bottom layer of agar and soaked in 50 mM l-lysine in PBS (pH 7.4) at 4°C for 4 h, fixed in 4% formaldehyde–50 mM l-lysine–PBS (pH 7.4) at 4°C for 4 h, and then incubated in the fixative overnight at 37°C. Fixed colonies were washed twice in PBS and dehydrated through a series of ethanol washes (25%, 50%, 70%, and 95% ethanol in PBS and 3 times with 100% ethanol) for 1 h each and then cleared by means of three 1-h washes in Histo-Clear-II (National Diagnostics HS-202). Cleared colonies were infiltrated with paraffin wax (Electron Microscopy Sciences; Fisher Scientific 50-276-89) at 55°C twice for 2 h each. Infiltrated colonies were solidified by overnight incubation at 4°C. Sections were cut perpendicularly to the base of the colony in 10-µm slices using an automatic microtome (Thermo Fisher Scientific 905200ER), floated over a water bath at 45°C, collected onto slides, and air-dried overnight. Dried slides were heat-fixed on a hotplate at 45°C for 1 h and then rehydrated to PBS by reversing the dehydration steps listed above. Sections were then immediately mounted beneath a coverslip in Tris-buffered DAPI (4′,6-diamidino-2-phenylindole)–Fluoro-Gel (Electron Microscopy Sciences; Fisher Scientific 50-246-93). Differential interference contrast (DIC) and fluorescent confocal images were captured for at least three biological replicates of each strain using an LSM700 confocal microscope (Zeiss).

### RNA-seq analysis.

Δ*phz* (45) colonies were grown on filter membranes (0.2-μm pore size, 25-mm diameter; Whatman) placed on 1% tryptone, 1.5% agar at 25°C for 76 h. Colonies were treated with RNAprotect bacterial reagent (Qiagen), samples were excised by microscopic laser dissection, and RNA was extracted using the RNeasy plant minikit (Qiagen). RNA samples were processed by Genewiz, including rRNA depletion and dUTP incorporation for strand-specific sequencing (Illumina HiSeq 2500 platform). Sixteen fastq files were mapped to the reference PA14 genome using Bowtie2 (46) with an ∼97% success rate to generate SAM (sequence alignment map) files. SAM files containing ∼2 × 108 reads in total were merged, sorted, and indexed with SAMtools (47). Read coverage was visualized with Integrative Genomics Viewer (IGV) software (48).

### Preparation of synthetic SCFM and derivatives.

Cystic fibrosis sputum medium (SCFM) was prepared as described previously (11). SCFM contains the following ingredients: 2.28 mM NH_4_Cl, 14.94 mM KCl, 51.85 mM NaCl, 10 mM MOPS, 1.3 mM NaH_2_PO_4_, 1.25 mM Na_2_HPO_4_, 0.348 mM KNO_3_, 0.271 mM K_2_SO_4_, 1.754 mM CaCl_2_, 0.606 mM MgCl_2_, 0.0036 mM FeSO_4_, 3 mM d-glucose, 9.3 mM sodium l-lactate, 0.827 mM l-aspartate, 1.072 mM l-threonine, 1.446 mM l-serine, 1.549 mM l-glutamate·HCl, 1.661 mM l-proline, 1.203 mM glycine, 1.78 mM l-alanine, 0.16 mM l-cysteine·HCl, 1.117 mM l-valine, 0.633 mM l-methionine, 1.12 mM l-isoleucine, 1.609 mM l-leucine, 0.802 mM l-tyrosine, 0.53 mM l-phenylalanine, 0.676 mM l-ornithine·HCl, 2.128 mM l-lysine·HCl, 0.519 mM l-histidine·HCl, 0.013 mM l-tryptophan, and 0.306 mM l-arginine·HCl. Depending on the solubility of the various salts, concentrations of their stock solutions ranged from 0.2 M to 1 M. Stock concentrations for d-glucose and sodium l-lactate were 1 M and for amino acid stocks 0.1 mM. No stock solution was prepared for l-tyrosine or l-tryptophan due to poor solubility. The pH of SCFM was adjusted to 6.5 with KOH and sterilized by filtration (Thermo Scientific Nalgene Rapid-Flow). ASMDM was prepared as previously described (26) by supplementing SCFM with 10 mg/ml bovine serum albumin, 10 mg/ml mucin from porcine stomach, and 1.4 mg/ml herring sperm DNA. SCFM2 was prepared by supplementing SCFM with 5 mg/ml mucin from porcine stomach, 100 µg/ml 1,2-dioleoyl-*sn*-glycero-3-phosphocholine (DOPC), 300 µM *N*-acetyl-d-glucosamine, and 600 µg/ml herring sperm DNA as previously described (27).
