# Supplementary material for: The Pseudomonas aeruginosa Complement of Lactate Dehydrogenases Enables Use of d- and l-Lactate and Metabolic Cross-Feeding
Source: mBio. 2018 Sep 11;9(5):e00961-18. doi: 10.1128/mBio.00961-18 (PMC6134097; doi:10.1128/mBio.00961-18)
Supplement: TABLE S1 [file mbo004184061st1.pdf]

TABLE S1 Strains and plasmids used in this study.

Strains/plasmids	Number	Used in figures	Characteristics	Source or reference
Pseudomonas aeruginosa				
PA14		3, 4, 5, 6, S3, S4, S5	Clinical isolate UCBPP-PA14	(1)
PA14 Δ*ldhA*	LD2729	3, 5, S3	PA14 with a deletion *ldhA*	This study
PA14 Δ*lldDE*	LD2735	3, 4, 5, S3, S4, S5	PA14 with a deletion *lldD-E*	This study
PA14 Δ*lldA*	LD2844	3, 4, 5, S3, S5	PA14 with a deletion *lldA*	This study
PA14 Δ*lldDE* Δ*lldA*	LD2759	3, 4, S5	PA14 with deletions of *lldD-E* and *lldA*	This study
PA14 Δ*lldDE* Δ*lldA ::lldDE*	LD2904	3	PA14 Δ*lldDE* Δ*lldA* with complementation of *lldD-E*	This study
PA14 *attB::*MCS-*gfp*	LD2820	2, 7, S2, S4	PA14 with MCS-*gfp* inserted at the *attB* site using pLD2722 (pSEK103)	This study
PA14 *attB::ldhAp-gfp*	LD3095	S2	PA14 with *ldhAp-gfp* inserted at the *attB* site using pLD2814 (pSEK103-*gacSp*)	This study
PA14 *attB::gacSp-gfp*	LD2815	5, 7, S2	PA14 with *gacSp-gfp* inserted at the *attB* site using pLD2814 (pSEK103-*gacSp*)	This study
PA14 *attB::lldPp-gfp*	LD2798	2, 4, 5, 6, 7, S4	PA14 with *lldPp-gfp* inserted at the *attB* site using pLD2797 (pSEK103-*lldPp*)	This study
PA14 *attB::lldAp-gfp*	LD2868	2, 4, 5, 6	PA14 with *lldAp-gfp* inserted at the *attB* site using pLD2867 (pSEK103-*lldAp*)	This study
Escherichia coli				
UQ950	LD44		*E. coli* DH5α λ(*pir*) strain for cloning; F^-Δ(*argF-lac*) 169φ80d*lacZ58*(ΔM15) *glnV44*(AS) *rfbD1 gyrA96*(Nal^R) *recA1 endA1 spoT thi-1 hsdR17 deoR* λpir^+	D. Lies
BW29427	LD661		Donor strain for biparental conjugation; *thrB1004 pro thi rpsL hsdS lacZ* ΔM15RP4–1360 Δ(*araBAD*)*567* Δ*dapA1341*::[*erm pir*(wt)]	B. Wanner
S17-1	LD2901		Str^R , Tp^R , F^- RP4-2-Tc::Mu *aphA*::Tn7 *recA* λpir lysogen	(2)
Saccharomyces cerevisiae				
InvSc1	LD622		*MATa/MATα leu2/leu2 trp1-289/trp1-289 ura3-52/ura3-52 his3-Δ1/his3-Δ1*	Invitrogen
Plasmids				
pMQ30	LD621		Yeast-based allelic-exchange vector; *sacB*$^+$, CEN/ARSH, $URA3^+$, Gm^R	(3)
pLD2728 (pMQ30-Δ*ldhA*)	LD2728		Δ*ldhA* flanking fragments introduced into pMQ30 by gap repair cloning in yeast strain InvSc1	This study
pLD2734 (pMQ30-Δ*lldDE*)	LD2734		Δ*lldDE* flanking fragments introduced into pMQ30 by gap repair cloning in yeast strain InvSc1	This study
pLD2758 (pMQ30-Δ*lldA*)	LD2758		Δ*lldA* flanking fragments introduced into pMQ30 by gap repair cloning in yeast strain InvSc1	This study
pLD2903 (pMQ30-*lldDE*-comp)	LD2903		*lldDE* complementation flanking fragments intro-duced into pMQ30 by gap repair cloning in yeast strain InvSc1	This study

pYL122	LD742		Amp^R *rhlA-gfp* transcription fusion in mini-CTX-*lacZ*	(4)
pUC18-mini-Tn7			Amp^R ColE1 replicon mini-Tn7 base vector	(5)
pLD844 (pSEK101)	LD844		*rhlA* promoter of pYL122 was removed (XhoI and EcoRI) and replaced with a multiple cloning site (MCS) from pUC18-mini-Tn7	(6)
pLD2722 (pSEK103)	LD2722		*aacC1* (gentamicin resistance cassette) from pMQ30 was inserted at the BspDI site of pLD844	(6)
pLD3094 (pSEK103-*ldhAp*)	LD3094		494 bp of *ldhA* promoter sequence inserted at the MCS (SpeI and EcoRI) of pLD2722	This study
pLD2814 (pSEK103-*gacSp*)	LD2814		297 bp of *gacS* promoter sequence inserted at the MCS (SpeI and XhoI) of pLD2722	This study
pLD2797 (pSEK103-*lldPp*)	LD2797		324 bp of *lldP* promoter sequence inserted at the MCS (SpeI and XhoI) of pLD2722	This study
pLD2867 (pSEK103-*lldAp*)	LD2867		356 bp of *lldA* promoter sequence inserted at the MCS (SpeI and XhoI) of pLD2722	This study
pFLP2	LD743		Site-specific excision vector with cI857-controlled FLP recombinase; *sacB*$^+$, Amp^R	(7)

1. Rahme LG, Stevens EJ, Wolfort SF, Shao J, Tompkins RG, Ausubel FM. 1995. Common virulence factors for bacterial pathogenicity in plants and animals. Science 268:1899–1902.

2. Teng F, Murray BE, Weinstock GM. 1998. Conjugal transfer of plasmid DNA from *Escherichia coli* to enterococci: A method to make insertion mutations. Plasmid 39:182–186.

3. Shanks RMQ, Caiazza NC, Hinsa SM, Toutain CM, O'Toole GA. 2006. *Saccharomyces cerevisiae*-based molecular tool kit for manipulation of genes from gram-negative bacteria. Appl Environ Microbiol 72:5027–5036.

4. Lequette Y, Greenberg EP. 2005. Timing and localization of rhamnolipid synthesis gene expression in *Pseudomonas aeruginosa* biofilms. J Bacteriol 187:37–44.

5. Choi K-H, Gaynor JB, White KG, Lopez C, Bosio CM, Karkhoff-Schweizer RR, Schweizer HP. 2005. A Tn7-based broad-range bacterial cloning and expression system. Nat Methods 2:443–448.

6. Lin Y-C, Sekedat MD, Cornell WC, Silva GM, Okegbe C, Price-Whelan A, Vogel C, Dietrich LEP. 2018. Phenazines regulate Nap-dependent denitrification in *Pseudomonas aeruginosa* biofilms. J Bacteriol JB.00031-18.

7. Hoang TT, Karkhoff-Schweizer RR, Kutchma AJ, Schweizer HP. 1998. A broad-host-range Flp-FRT recombination system for site-specific excision of chromosomally-located DNA sequences: application for isolation of unmarked *Pseudomonas aeruginosa* mutants. Gene 212:77–86.
