# Supplementary material for: The Pseudomonas aeruginosa Complement of Lactate Dehydrogenases Enables Use of d- and l-Lactate and Metabolic Cross-Feeding
Source: mBio. 2018 Sep 11;9(5):e00961-18. doi: 10.1128/mBio.00961-18 (PMC6134097; doi:10.1128/mBio.00961-18)
Supplement: TABLE S2 [file mbo004184061st2.pdf]

TABLE S2 Primers used in this study.

Number	Name	Sequence	Used for
1087	attB::gfp-inCheck1	AGGGCCAATCGATAGAGTTT	To verify the genotype of gfp reporter strains
1088	attB::gfp-inCheck2	TCTTCGTGATCTGAAGCCATT	To verify the genotype of gfp reporter strains
1089	attB::gfp-outCheck1	TAGAAGAACAGGCGGACGAT	To verify the genotype of gfp reporter strains
1090	attB::gfp-outCheck2	AGCATCATCGGTACCCAGTC	To verify the genotype of gfp reporter strains
2072	ΔldhA-yeast1	ggaattgtgagcggataacaatttcacacaggaaacagct AAGAGCTGAGCCAGTCGTTC	To make pLD2728 (pMQ30-Δ*ldhA*) by gap repair cloning in yeast strain InvSc1
2073	ΔldhA-yeast2	atgttgtccagggtggtgtc CTGGAAGCTCTCGCTGTCGTAG	To make pLD2728 (pMQ30-Δ*ldhA*) by gap repair cloning in yeast strain InvSc1
2074	ΔldhA-yeast3	ctacgacagcgagagcttccag GACACCACCCTGGACAACAT	To make pLD2728 (pMQ30-Δ*ldhA*) by gap repair cloning in yeast strain InvSc1
2075	ΔldhA-yeast4	aggcaaattctgttttatcagaccgcttctgcgttctgat CTAGGGACGTGGTGGTCAAG	To make pLD2728 (pMQ30-Δ*ldhA*) by gap repair cloning in yeast strain InvSc1
2076	ΔldhA-check1	CTCGACAGCCTCCAGGAA	To verify the genotype of Δ*ldhA* strains
2077	ΔldhA-check2	CAGGGTGTAACGGAAATCGT	To verify the genotype of Δ*ldhA* strains
2078	ΔldhA-check3	CTGGACAAGGCCATCGAG	To verify the genotype of Δ*ldhA* strains
2079	ΔldhA-check4	CAGCCACGACAACAGAAAGA	To verify the genotype of Δ*ldhA* strains
2088	ΔlldDE-yeast1	ggaattgtgagcggataacaatttcacacaggaaacagct CCTACAGCTTCGGCGAGAT	To make pLD2734 (pMQ30-Δ*lldDE*) by gap repair cloning in yeast strain InvSc1
2089	ΔlldDE-yeast2	caccaggtagaccacccc GTAGTCGGTGGAAGCGGAAAT	To make pLD2734 (pMQ30-Δ*lldDE*) by gap repair cloning in yeast strain InvSc1
2090	ΔlldDE-yeast3	atttccgcttccaccgactac GGGGTGGTCTACCTGGTG	To make pLD2734 (pMQ30-Δ*lldDE*) by gap repair cloning in yeast strain InvSc1
2091	ΔlldDE-yeast4	aggcaaattctgttttatcagaccgcttctgcgttctgat TCCGGCGGATAGAAATAGAA	To make pLD2734 (pMQ30-Δ*lldDE*) by gap repair cloning in yeast strain InvSc1
2092	ΔlldDE-check1	ACCCTGTTCCTCAAGGTCTG	To verify the genotype of Δ*lldDE* strains
2093	ΔlldDE-check2	GTCGTGACGTAGGGCGAATA	To verify the genotype of Δ*lldDE* strains
2094	ΔlldDE-check3	GCATGCTGGTGCACTGAC	To verify the genotype of Δ*lldDE* strains
2095	ΔlldDE-check4	CAGGCGGTAGACGTACTGGT	To verify the genotype of Δ*lldDE* strains
2100	ΔlldA-yeast1	ggaattgtgagcggataacaatttcacacaggaaacagct ATTTCCGTTTTCCTTCCATC	To make pLD2758 (pMQ30-Δ*lldA*) by gap repair cloning in yeast strain InvSc1
2101	ΔlldA-yeast2	tatcgcgggtagcttcccgg GTCTTCGATATCGGTGATGACG	To make pLD2758 (pMQ30-Δ*lldA*) by gap repair cloning in yeast

			strain InvSc1
2102	ΔlldA-yeast3	cgtcatcaccgatatcgaagac CCGGGAAGCTACCCGCGATA	To make pLD2758 (pMQ30-Δ*lldA*) by gap repair cloning in yeast strain InvSc1
2103	ΔlldA-yeast4	aggcaaattctgttttatcagaccgcttctgcgttctgat TAACCTGGCAGAACTGAACG	To make pLD2758 (pMQ30-Δ*lldA*) by gap repair cloning in yeast strain InvSc1
2104	ΔlldA-check1	GACGCGGTATCCCTGATCT	To verify the genotype of Δ*lldA* strains
2105	ΔlldA-check2	ACCTGCTGGAAAGCTTCGAC	To verify the genotype of Δ*lldA* strains
2106	ΔlldA-check3	GACTGAAACGGCGGAATTT	To verify the genotype of Δ*lldA* strains
2107	ΔlldA-check4	CTCATCGGACTGAAGGGAGA	To verify the genotype of Δ*lldA* strains
2340	PldhA-F	acgtacgtacactagtGGCATGGACGACTACCTGAC	To make pLD3094 (pSEK103-*ldhAp*) by restriction-ligation
2341	PldhA-R	acgtacgtacgaattcGGTCAGAGTTCGCTGGAGTC	To make pLD3094 (pSEK103-*ldhAp*) by restriction-ligation
2179	PgacS-F	acgtacgtacactagtACCAGGTCACCTCGAAACC	To make pLD2814 (pSEK103-*gacSp*) by restriction-ligation
2180	PgacS-R	acgtacgtacctcgagACGTCTCTCCGTCGAGCCAGAT	To make pLD2814 (pSEK103-*gacSp*) by restriction-ligation
2146	PlldP-F	acgtacgtacactagtCGACACCCTTACCCGAAGT	To make pLD2797 (pSEK103-*lldPp*) by restriction-ligation
2147	PlldP-R	acgtacgtacctcgaGGGTTGGCTCCCTAATTGTT	To make pLD2797 (pSEK103-*lldPp*) by restriction-ligation
2203	PlldA-F	acgtacgtacactagtTGCTCGATTTGGGCATGA	To make pLD2867 (pSEK103-*lldAp*) by restriction-ligation
2204	PlldA-R	acgtacgtacctcgagGCAGTCCACTCCTTCGGG	To make pLD2867 (pSEK103-*lldAp*) by restriction-ligation
2213	lldDE-comp-yeast1	ggaattgtgagcggataacaatttcacacaggaaacagctCCTACAGCTTCG GCGAGAT	To make pLD2903 (pMQ30-*lldDE*-comp) by gap repair cloning in yeast strain InvSc1
2214	lldDE-comp-yeast2	ACGGTCTCGAGAAAGGGAAT	To make pLD2903 (pMQ30-*lldDE*-comp) by gap repair cloning in yeast strain InvSc1
2215	lldDE-comp-yeast3	CGCCAGAAAAGCCTGAAA	To make pLD2903 (pMQ30-*lldDE*-comp) by gap repair cloning in yeast strain InvSc1
2216	lldDE-comp-yeast4	CGGCCTTTTCCAGCAGAC	To make pLD2903 (pMQ30-*lldDE*-comp) by gap repair cloning in yeast strain InvSc1
2217	lldDE-comp-yeast5	GGCCACGGGTGGTCTATC	To make pLD2903 (pMQ30-*lldDE*-comp) by gap repair cloning in yeast strain InvSc1
2218	lldDE-comp-yeast6	aggcaaattctgttttatcagaccgcttctgcgttctgatTCCGGCGGATAGAA ATAGAA	To make pLD2903 (pMQ30-*lldDE*-comp) by gap repair cloning in yeast strain InvSc1
